# Lack of controlled studies investigating the risk of postpartum haemorrhage in cesarean delivery after prior use of oxytocin: a scoping review

**DOI:** 10.1186/s12884-017-1584-1

**Published:** 2017-11-29

**Authors:** Karin Bischoff, Monika Nothacker, Cornelius Lehane, Britta Lang, Joerg Meerpohl, Christine Schmucker

**Affiliations:** 1 Cochrane Germany, Medical Center – University of Freiburg, Faculty of Medicine, University of Freiburg, Freiburg, Germany; 2 AWMF-Institute for Medical Knowledge Management (IMWi), Karl-von-Frisch-Street 1, 35043 Marburg, Germany; 3 Department of Anesthesiology and Critical Care, University Heart Center Freiburg-Bad Krozingen, Medical Center - University of Freiburg, Freiburg, Germany

**Keywords:** Oxytocin, Uterotonics, Postpartum haemorrhage, Intrapartum cesarean section

## Abstract

**Background:**

Postpartum haemorrhage (PPH) is a major cause of maternal mortality and morbidity worldwide. Experimental and clinical studies indicate that prolonged oxytocin exposure in the first or second stage of labour may be associated with impaired uterine contractility and an increased risk of atonic PPH. Therefore, particularly labouring women requiring cesarean delivery constitute a subset of patients that may exhibit an unpredictable response to oxytocin. We mapped the evidence for comparative studies investigating the hypothesis whether the risk for PPH is increased in women requiring cesarean section after induction or augmentation of labour.

**Methods:**

We performed a systematic literature search for clinical trials in Medline, Embase, Web of Science, and the Cochrane Library (May 2016). Additionally we searched for ongoing or unpublished trials in clinicaltrials.gov and the WHO registry platform. We identified a total of 36 controlled trials investigating the exogenous use of oxytocin in cesarean section. Data were extracted for study key characteristics and the current literature literature was described narratively.

**Results:**

Our evidence map shows that the majority of studies investigating the outcome PPH focused on prophylactic oxytocin use compared to other uterotonic agents in the third stage of labour. Only 2 dose-response studies investigated the required oxytocin dose to prevent uterine atony after cesarean delivery for labour arrest. These studies support the hypotheses that labouring women exposed to exogenous oxytocin require a higher oxytocin dose after delivery than non-labouring women to prevent uterine atony after cesarean section. However, the study findings are flawed by limitations of the study design as well as the outcome selection. No clinical trial was identified that directly compared exogenous oxytocin versus no oxytocin application before intrapartum cesarean delivery.

**Conclusion:**

Despite some evidence from dose-response studies that the use of oxytocin may increase the risk for PPH in intrapartum cesarean delivery, current research has not investigated the prepartal application of oxytocin in well controlled clinical trials. It was striking that most studies on exogenous oxytocin are focused on PPH prophylaxis in the third stage of labour without differing between the indications of cesarean section and hence the prepartal oxytocin status.

**Electronic supplementary material:**

The online version of this article (10.1186/s12884-017-1584-1) contains supplementary material, which is available to authorized users.

## Background

Postpartum haemorrhage (PPH) is a major cause of maternal mortality and morbidity worldwide [1–4]. Approximately 36% of all lower segment cesarean deliveries and between 6 and 14% of spontaneous deliveries are complicated by PPH, depending on the definition used and the population studied [5–7]. Although global mortality from PPH is falling, its incidence is increasing in several high resource settings [8, 9]. In the United States, between 1994 and 2006, the rate of atonic PPH increased by 160% among women undergoing cesarean section after induction of labour and 130% among women undergoing non-induced cesarean section [10]. This increase may be explained by the increasing rates of cesarean sections and associated complications caused by general anesthesia, operative techniques or complications such as placental abruption leading to an emergency cesarean section [8, 11–14], but also due to an extensive use of uterotonics to induce or augment labour [7, 14].

The most widely used uterotonic drug for augmenting labour or to maintain uterine contractility during labour is oxytocin [15, 16]. Oxytocin, which was first synthetically synthesized in 1953 is also the first choice in the prevention and treatment of uterine atony [17]. When given in low-dose, oxytocin induces rhythmic uterine contractions which are indistinguishable in frequency, duration and strength from contractions observed during spontaneous labour [18, 19]. On the other hand, oxytocin for inducing or augmenting labour may desensitise the oxytocin receptors, thereby impairing oxytocin’s post-delivery effects on uterine contractility [18]. For example, Balki et al. showed in different in vitro studies that pretreatment with oxytocin decreases oxytocin-induced myometrial contractions in pregnant humans and animals [20–22]. In addition, Grotegut et al. conducted a retrospective study on women who experienced severe PPH and found that these women received significantly greater amounts of oxytocin during labour compared with women without PPH, suggesting that prolonged pre- or intrapartum exposure to oxytocin might lead to decreased drug efficacy [23]. Similarly, Belghiti et al. investigated in a population-based case-control study the association between the level of oxytocin exposure during labour and the risk of severe atonic PPH [24, 25]. The study showed that oxytocin exposure during labour appears to be an independent risk factor for severe PPH in woman with spontaneous labour who did not receive prophylactic oxytocin after delivery (odds ratio after adjustment for all potential confounders [adjusted OR]: 1.8, 95% CI 1.3 to 2.6). Thereby, the strength of the association increased with the amount of oxytocin infused during labour [24, 25].

Considering that PPH rates are higher in cesarean section compared to spontaneous labour [7], particularly labouring women requiring cesarean delivery may constitute a subset of patients that may exhibit a negative response to exogenous oxytocin [14–16]. Therefore, we mapped the current literature for controlled clinical studies investigating the risk of atonic PPH in labouring women requiring cesarean delivery (intrapartum cesarean section) after oxytocin use for labour induction or augmentation in comparison to no exogenous oxytocin.

## Methods

### Sources

We adhered to the Preferred Reporting Items for Systematic reviews and Meta-Analyses (PRISMA) protocol for identifying, screening and eligibility of studies to conduct the present research work [see Additional file 1].

We searched for controlled clinical studies investigating exogenous oxytocin in cesarean delivery, irrespective of delivery indication, age, comorbidity or woman’s parity. Primary maternal outcomes, we were interested in, included PPH rates, adverse effects or the need for additional uterotonics. In addition we collated any neonatal outcomes reported. A review protocol can be accessed from the corresponding author (CS).

Published studies were identified from searches of electronic databases. We searched Medline (OvidSP), Embase, Web of Science, the Cochrane library from inception until May 2016. The search strategy was based on combinations of medical subject headings (MeSH) and keywords and was not restricted to specific languages. The search strategy used in Medline (OvidSP) is presented in an additional file [see Additional file 2]. Search strategies for other databases were modified to meet the requirements of each database. The searches were supplemented by screening the bibliographies of relevant studies and systematic reviews. Potential ongoing studies were identified in the International Clinical Trials Registry Platform WHO (http://www.who.int/ictrp/en/) and the Register for Clinical Trials (http://clinicaltrials.gov/).

### Study selection

One author (KB) screened the titles and abstracts of all reports identified by electronic searches. We obtained full-text copies of all potentially relevant articles and 2 reviewers (KB, CS) assessed them for inclusion. These authors also independently carried out data extraction for key characteristics of the study, participant and intervention.

### Data analysis (mapping the evidence)

First, study characteristics such as country, study design, details of the intervention and control intervention, time point of drug application, week of gestation, sample size, age and indication for cesarean section were extracted and tabulated. Second, we looked whether the given PPH rates were stratified after the indication for cesarean delivery and/or when only women with intrapartum cesarean section were included whether the results were stratified after the use of oxytocin versus no oxytocin before surgery. By considering key characteristics of all studies investigating oxytocin in cesarean section, evidence gaps for a subset of women could be identified and a narrative description of the current literature provided.

## Results

### Published studies

**Fig. 1:**
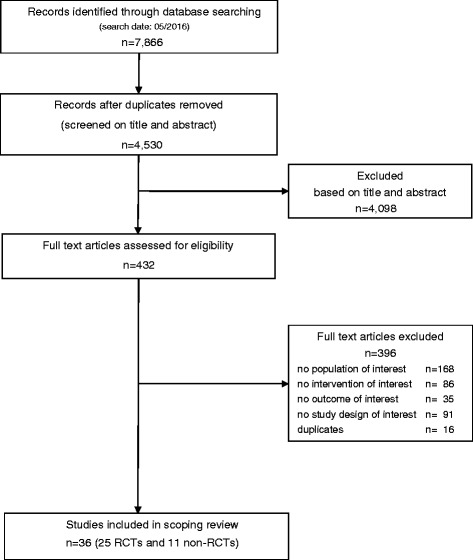
PRISMA flow diagram: Results of the bibliographic literature search in Medline, Embase, Web of Science, and the Cochrane Library in May 2016 (as published by *Moher D* et al. *in BMJ 2009;339:b2535*). RCT: randomized controlled trial

We identified 7866 titles and abstracts; for 432 of these, the full text was evaluated. Figure 1 outlines the screening and selection process of the articles. Table 1 presents the key characteristics of the 36 controlled trials investigating the risk of PPH after the use of exogenous oxytocin in cesarean section: 23 randomized controlled trials (RCTs) (including 4084 patients), 2 comparative dose-response studies (including 100 patients) and 11 non-RCTs (including 3896 patients).

**Table 1:** Studies Included in Scoping Review

	Study & Country	Study type	Intervention (I1) & Control (I2)	Time point of drug application	Population				Follow-up & beginning and end of study
					Week of gestation (mean ± SD)	Women (n)	Age (mean ± SD; median [range])	Indication	
Comparative dose-response studies	Balki 2006 [26] Canada	RCT	“the dose of oxytocin for each patient was determined by the response of the previous patient (±0,5 IU) to the drug, according to a biased coin up- and-down sequential allocation scheme to cluster doses close to the minimum effective dose (ED_90_)”^a^	“immediately upon delivery of the anterior shoulder of the infant”	39.9 ± 1.1	total: 30	32.7 ± 4.4	CS for labour arrest “augmentation with iv oxytocin for a minimum of 2 h”	48 h 03/04–12/04
	Lavoie 2015 [27] US	RCT	“dose-response study using a 9:1 biased-coin sequential allocation method to estimate the ED_90_ of an infusion of prophylactic oxytocin in women undergoing CS with neuraxial anesthesia. The starting infusion rate was 18 IU/h, with an incremental dose of 2 IU/h.”	“immediately after the umbilical cord was clamped”	I1: 40 (40–41) I2: 39 (39–39)	I1: 32 I2: 38	I1: 33 (28–35) I2: 33 (31–36)	I1:“labouring group: women scheduled for intrapartum CS after prior exposure to exogenous oxytocin”^b^ I2:“non-labouring group: women scheduled for CS”	24 h 08/12–06/13
Different Oxytocin doses	Munn 2001 [28] US	RCT	I1: Oxytocin 10 IU, *iv*, 30 min I2: Oxytocin 80 IU, *iv*, 30 min +prn Oxytocin for labour induction;^c^ after delivery in the postanesthesia care unit, all patients received oxytocin, 20 IU, *iv*, 8 h	“after cord clamping”	I1: 37 ± 4.3 I2: 37 ± 4.9	I1: 163 I2: 158	I1: 25 ± 6.0 I2: 25 ± 6.1	labour before CS^d^	1 day 01/97–11/99
	Ayedi 2011 [55] Tunisia	RCT	I1: Oxytocin 2 IU (in 5 ml volume), *iv,* single-dose, 5–10 s I2: Oxytocin 5 IU (in 5 ml volume), *iv,* single-dose, 5–10 s +prn uterotonics	“after delivery of the baby and cord clamping”	≥37	total: 60	–	–	–
	Chou 2015 [56] Malaysia	RCT	I1: Oxytocin 40 IU (in 500 ml NaCl), *iv,* over 6 h I2: Oxytocin 80 IU (in 500 ml NaCl), *iv,* over 6 h +all P. were given an oxytocin bolus of 5 IU	“post-delivery”	–	–	–	–	- 11/12–05/13
	Khan 2012 [32] India	RCT	I1: Oxytocin 10 IU (in 500 ml NaCl), *iv,* over 1 h I2: Oxytocin 20 IU (in 500 ml NaCl), *iv,* over 1 h +prn uterotonics	–	–	total: 200	–	emergency	24 h study duration: 6 mos
	Kintu 2012 [54] Uganda	RCT	I1: Oxytocin 2.5 IU, no further information I2: Oxytocin 10 IU, no further information +prn uterotonics	“following cord clamping”	–	total: 380	–	emergency and elective	24 h -
	Lee 2014 [48] US	Non-RCT (retrospective)	I1: Oxytocin 10 IU, *iv* Infusion was continued until P. transfer to the recovery room I2: Oxytocin 30 IU, *iv,* over 1 h	“following cord clamping”	–	I1: 483 I2: 418	I1: 32 ± 6 I2: 33 ± 6	–	- I1: 09/08–11/08 I2: 11/08–01/09
	Pursche 2012 [42] Germany	Non-RCT (retrospective)	I1: Oxytocin 3 IU, *iv*, single-dose + continuously 3 IU oxytocin, *iv*, infusion rate 100 ml/h I2: Oxytocin continuously 3 IU oxytocin, *iv*, infusion rate at least 120 ml/h	“postpartum”	I1: 37.9 I2: 37.9	I1: 228 I2: 227	–	elective (*n* = 71) and during labour (*n* = 384)^e^	4 h 01/11–12/11
	McClune 2011 [44] UK	Non-RCT (retrospective)	Different doses oxytocin, not administered uniformly^f^ + prn uterotonics	“given at cord clamping”	–	total: 50	–	–	- 08/10–09/10
Application schema	Mangla 2012 [59] India	RCT	I1: Oxytocin 20 IU, *iv* I2: Oxytocin 5 IU, *imy* I3: Oxytocin 5 IU, *imy* No information about additional uterotonics	I1 + I2: “after separation of placenta” I3: “before separation of placenta”	–	I1: 50 I2: 50 I3: 50	–	–	1 h -
Oxytocin vs other uterotonics time-dependent	Adefuye 2012 [50] Nigeria	RCT	I1: Oxytocin 20 IU, *iv* I2: Misoprostol 2 × 400 μg, *sublingual* +prn uterotonics intraop	I1: “after delivery” I2: “before delivery”	I1: 38.0 ± 0.2 I2: 38.1 ± 0.3	I1: 50 I2: 50	I1: 28.7 ± 0.7 I2: 29.9 ± 0.8	elective (28%) and emergency (72%)^g^	24 h 04/09–03/11
	Chaudhuri 2010 [60] India	RCT	I1: Oxytocin 8 × 5 IU, *iv* + placebo, *rectal* I2: Misoprostol 4 × 200 μg, *rectal* + 8 placebo, *iv* +prn uterotonics	I1: “after delivery” I2: “before incision”	I1: 39.2 ± 1.4 I2: 39.5 ± 1.7	I1: 100 I2: 100	I1: 24.3 ± 5.0 I2: 24.0 ± 3.4	elective and emergency^h^ without risk factors for PPH	24 h 12/07–06/09
Oxytocin vs placebo	King 2010 [49] Canada	RCT	I1: Oxytocin 5 IU, *iv*, über 30 s, single-dose I2: Placebo NaCl, *iv*, 30 s, single-dose +routinely oxytocin (40 IU, *iv*, 30 min) followed by oxytocin (20 IU) immediately after study drug +prn uterotonics	“as soon as the umbilical cord was clamped”	I1: 37.0 ± 3.0 I2: 38.0 ± 2.0	I1: 75 I2: 75	I1: 34 ± 5 I2: 34 ± 6	elective (*n* = 76) and non-elective (*n* = 67) at least one risk factor for PPH	24 h 11/05–10/06
Oxytocin vs other uterotonics	Attilakos 2010 [34] UK	RCT	I1: Oxytocin 5 IU, *iv*, 30–60 s I2: Carbetocin 100 μg, *iv*, 30–60 s +prn uterotonics (Oxytocin for PPH prophylaxis or treatment)	“after the birth of the baby”	≥37	I1: 189 I2: 188	I1: 32 (18–44) I2: 32 (18–42)	elective (60%) and emergency (40%) 1/3 showed risk factors for PPH	until discharged 11/06–07/07
	Borruto 2009 [35] Italy	RCT	I1: Oxytocin 10 IU, *iv*, 2 h I2: Carbetocin 100 μg, *iv*, single-dose +prn uterotonics	“immediately following placental delivery”	≥36	I1: 52 I2: 52	32.2 (22–41)	planned and emergency^i^ at least one risk factor for PPH	24 h 09/07–01/08
	Catanzarite 1990 [47] US	RCT	I1: Oxytocin 20 IU, *imy* I2: Carboprost tromethamine 125 μg, *imy* +routinely oxytocin (20 IU, *iv*) after placental delivery	“immediately following placental delivery”	–	I1: 21 I2: 25	I1: 24.5 I2: 24.1	planned and emergency^j^	3 days -
	Chaudhuri 2014 [33] India	RCT	I1: Oxytocin 8 × 5 IU, *iv*, 12 h + placebo *rectal* I2: Misoprostol 4 × 200 μg, *rectal* + placebo *iv*, 12 h +routinely oxytocin, not later than 1 min after delivery	“at the end of the operation”	I1: 38.8 ± 1.2 I2: 39.0 ± 1.1	I1: 96 I2: 96	I1: 23.2 ± 3.7 I2: 23.5 ± 4.5	emergency without risk factor for PPH	24 h 05/11–05/12
	Lapaire 2006 [36] Switzerland	RCT	I1: Oxytocin 20 IU, *iv*, 8 h + placebo *oral* I2: Misoprostol 800 μg, *oral* + placebo *iv*, 8 h +routinely oxytocin (5 IU, *iv*) after cord clamping	“immediately after cord clamping”	I1: 38.7 ± 1.3 I2: 38.6 ± 1.3	I1: 28 I2: 28	I1: 31.2 ± 5.1 I2: 32.2 ± 6.5	elective and indicated^k^ low risk for PPH	48 h 01/99–02/02
	Lokugamage 2001 [37] UK	RCT	I1: Oxytocin 10 IU, *iv*, single-dose + placebo, *oral* I2: Misoprostol 500 μg, *oral* + placebo *iv*, single-dose +prn uterotonics	“immediately after the delivery of the baby”	I1: 38.3 ± 1.1 I2: 36.7 ± 8.9	I1: 20 I2: 20	I1: 31.4 ± 5.5 I2: 32.3 ± 6.4	elective (n = 38) and emergency (*n* = 2) excluded: previous rupture of the uterus	3 days -
	Owonikoko 2011 [51] Nigeria	RCT	I1: Oxytocin 20 IU, *iv* I2: Misoprostol 400 μg, *sublingual* +prn Oxytocin *iv*	“immediately after extraction of the baby”	I1: 39.7 ± 1.9 I2: 38.7 ± 2.3	I1: 50 I2: 50	I1: 30.4 ± 4.8 I2: 31.5 ± 4.1	elective (35%)^l^ and emergency (65%)^m^	24 h 06/06–04/07
	Razali 2016 [30] Malaysia	RCT	I1: Oxytocin 10 IU, *iv*, single-dose I2: Carbetocin 100 μg, *iv*, single-dose +prn uterotonics	“after delivery”	“at term”	I1: 300 I2: 300	I1: 29.7 ± 4.3 I2: 29.5 ± 4.6	emergency^n^	24 h 14/08–09/12
	Vimala 2006 [61] India	RCT	I1: Oxytocin 20 IU, *iv*, 6 h I2: Mis +prn uterotonics	“immediatiely after delivery of the neonate”	I1: 38.7 ± 1.1 I2: 38.7 ± 1.2	I1: 50 I2: 50	I1: 26.3 ± 3.7 I2: 25.6 ± 5.1	elective (17%) and emergency (83%)^o^ without risk factors for PPH	24 h 08/04–04/05
	El Behery 2016 [29] Egypt	RCT	I1: Oxytocin 20 IU, *iv* I2: Carbetocin 100 μg, *iv*, 2 min +prn uterotonics	“after delivery of the infant preferably before placental removal”	I1: 38.2 ± 0.8 I2: 38.2 ± 1.2	I1: 90 I2: 90	I1: *n* = 90 I2: n = 90	emergency	24 h 01/13–06/14
	Whigham 2016 [31] Australia	RCT	I1: Oxytocin 5 IU, *iv* I2: Carbetocin 100 μg, *iv* +prn uterotonics	“immediately after birth of the baby”	I1: 40.0 ± 1.4 I2: 39.6 ± 1.5	I1: 53 I2: 59	I1: 28.9 ± 5.8 I2: 28.3 ± 5.9	non-elective and emergency^p^	- 08/12–02/13
	Alli 2013 [53] Nigeria	RCT	I1: Oxytocin 10 IU, *iv,* single-dose I2: Misoprostol 600 μg, *sublingual* +prn uterotonics	“immediately after delivery of the baby”	–	total: 100	–	–	–
	Begum 2015 [57] Bangladesh	RCT	I1: Oxytocin 20 IU (in 100 ml Dextrose NaCl), *iv* I2: Misoprostol 400 μg, *sublingual* +prn uterotonics	“soon after delivery”	“term pregnancy”	total: 100	–	–	–
	Pizzagalli 2015 [41] France	Non-RCT (prospective)	I1: Oxytocin 5 IU *iv*, followed by 10 IU *iv* during surgery, further infusions 10 IU 8 h postop I2: Carbetocin 100 μg *iv* I3: Oxytocin 5 IU *iv*, followed by 10 IU *iv* during surgery, further infusions 10 IU 8 h + Sulprostone	“during cord clamping”	≥37	I1: 282 I2: 262 I3: 258	–	CS before labour (*n* = 283) and unclear indication (*n* = 519)	24 h I1: 07/10–12/10 I2: 02/11–12/11 I3: 08/11–01/12
	Triopon 2010 [62] France	Non-RCT (retrospective)	I1: Carbetocin 100 μg, *iv* I2: Oxytocin 5 IU, *iv*	“after cord clamping”	I1: 38.2 ± 3.0 I2: 37.9 ± 3.2	I1: 155 I2: 155	I1: 30.6 ± 5.3 I2: 30.7 ± 5.4	elective and during labour	24 h I1: 04/09–07/09 I2: 03/07–06/07
	Demetz 2013 [46] France	Non-RCT (retrospective)	I1: Oxytocin 10 IU, *iv*, single-dose^q^ +3 h after delivery: 10 IU Oxytocin *iv* over 12 h I2: Carbetocin 100 μg, *iv*, single-dose +3 h after delivery: 10 IU Oxytocin *iv* over 12 h	“at the delivery”	I1: 35.5 ± 2.5 I2: 34.3 ± 3.1	I1: 24 I2: 39	I1: 31.6 ± 4.3 I2: 31.1 ± 5.8	planned (*n* = 31) and unclear (*n* = 32)	48 h I1: 08/09–01/10 I2: 02/10–07/10
	Testa 2013 [45] Italy	Non-RCT (retrospective)	I1: Oxytocin 10 IU, *iv*, 2 h I2: Carbetocin 100 μg, *iv*, single-dose	–	–	I1: 14 I2: 14	–	planned and emergency	24 h after drug application -
	Brzozowska 2015 [63] Polska	Non-RCT (retrospective)	I1: Oxytocin 10 IU, *im* I2: Carbetocin 100 μg, *iv* +prn uterotonics	–	I1: 38.3 ± 1.9 I2: 38.1 ± 1.9	I1: 140 I2: 139	I1: 30.5 ± 4.6 I2: 31.5 ± 5.1	–	- 03/14–10/14
Combination therapy	Koen 2016 [52] South Africa	RCT	I1: Oxytocin 2,5 IU, *iv*, single-dose + placebo *im* I2: Oxytocin + Ergometrine Placebo *iv* + Oxytocin 5 IU, *im* + Ergometrine 0.5 mg, *im* +routinely 10 IU Oxytocin	“after delivery of the neonate”	I1: 38.4 ± 2.2 I2: 38.5 ± 1.9	I1: 214 I2: 202	I1: 28.6 ± 6.0 I2: 28.9 ± 5.4	elective (36%) and emergency (64%)^r^	6–24 h 01/4–06/14
	Mahmud 2014 [58] Pakistan	Non-RCT (prospective)	I1: Oxytocin 10 IU *iv* I2: Oxytocin 10 IU *iv* + Ergometrine 0,25 mg *im*	“intraoperatively”	I1: 37.5 ± 2.0 I2: 38.0 ± 1.8	I1: 378 I2: 323	I1: 28.0 ± 3.5 I2: 29.0 ± 3.4	elective and emergency	6 h I1: 11/10–12/10 I2: 01/11–02/11
	Bayoumeu 2003 [38] France	Non-RCT (retrospective)	I1: Oxytocin 5 IU *iv (single dose)*, 10 IU *iv* 30 min, afterwards 25 IU *iv* 24 h I2: Oxytocin 5 IU *iv (single dose)*, 10 IU *iv* 30 min, afterwards 25 IU *iv* 24 h + Dinoprost/Sulprostone	“after clamping the last umbilical cord”	I1: 32.0 ± 2.7 I2: 32.8 ± 1.7	I1: 14 I2: 28	I1: 29.1 ± 3.2 I2: 30.2 ± 3.3	elective and emergency	48 h I1: 01/93–11/96 I2: 12/96–12/00
	Lourens 2007 [40] UK	Non-RCT (prospective)	I1: Oxytocin 5 IU iv, single-dose I2: Oxytocin 5 IU *iv*, single-dose + Ergometrine 0.5 mg *im*	Oxytocin: “after delivery” Ergometrine: “during abdominal closure”	I1: 38.0 ± 1.0 I2: 38.0 ± 1.0	I1: 158 I2: 107	–	elective (*n* = 120) and emergency (*n* = 145)	- I1: 02/06–03/06 I2: 04/06–06/06

^a^The dose of oxytocin for each woman was determined by the response of the previous woman to the drug, (…). If a woman did not respond adequately to the initial bolus of oxytocin, the initial dose for the next one was increased by 0.5 IU. If the woman responded to the initial bolus, the dose for the next one was decreased by 0.5 IU with a probability of 1/9; otherwise it remained unchanged. (…) . The starting dose of oxytocin was arbitrarily chosen as 0.5 IU

^b^CS due to labour dystocia, defined as arrest of dilation in first stage of labour or arrest of descent in second stage of labour

^c^Results were not reported separately for women with or without prepartal oxytocin administration

^d^Labour was defined as at least two contractions in 10 min and either an initial cervical dilation of at least 2 cm or progressive cervical change. Indications: non-reassuring FHR tracing (58%), arrest of labour (75%), abnormal lie (44%), other reasons (24%)

^e^Cardiotocography abnormalities (92), breech presentation (67), macrosomia (19), preeclampsie/HELLP (14), state after recesarean (22), twins/triplets (24/2), obstructed labour (29), fetal abnormalities/disease (22), maternale disease (54), placenta praevia/ bleeding (21)

^f^Initial bolus dose of oxytocin given at cord clamping and any subsequent bolus doses were recorded along with the concentration and rate of the maintenance oxytocin infusion if used and any other uterotonic drugs

^g^Indications: severe pregnancy induced hypertension/hypertensive disorders (20), uncontrolled diabetes mellitus (1), precious baby (bad obstetric history/pregnancy after infertility treatment) (2), fetal distress (33), abnormal lie (transverse/oblique lie) (3), malpresentation (face/brow) (5), dystocia (foeto-pelvic disproportion/occipito-posterior/transverse arrest) (23), macrosomia (3), breech (6), prolonged pregnancy (4)

^h^Indications: previous cs (51), prolonged pregnancy (failed induction) (23), malpresentation (10), preeclampsie (11), PROM (9), cephalopelvic disproportion (14), fetal distress (27), non-progress of labour (19), poor obstetric history (6), IUGR/oligohydramnios (7), elderly primigravida/infertility treated (13)

^i^Indications: previous cs (28), abnormal presentation (24), dystocia (18), FHR anomalies (16), umbilical cord prolapse (2), feto-pelvic disproportion (2), IUGR (2), fetal megalosomy (2), abruptio placentae (2), placenta previa (2), maternal disease (2), failed induction of labour (2), maternal request (2)

^j^Indications: elective, prior to labour (35), early labour (7), cephalopelvic disproportion (4)

^k^Indications: elective cs or breech presentation (31), malposition (1), twin pregnancy (1), repeated cs (5), ineffective induction of labour (2), ineffective induction of labour and infection (1), failure of labour to progress (1), hip dysplasia (1), IUGR (2), ankylosing spondylitis (1), macrosomia (2), placenta previa (2), Increased pressure not indicated (retinal disease, colposuspension) (3)

^l^Indication for elective cs: maternal, medical condition (HIV, herpes) (4), two previous cs (9), malpresentation (transverse lie, breech in primigravidae etc.) (6), maternal request for cs (4), fetal macrosomia (12)

^m^Indication for emergency: fetal distress (14), cephalopelvic disproportion (9), malpresentation in labour (9), failed vaginal birth after cs (20), failed induction of labour (4), severe oligohydramnios (9)

^n^Indication for emergency: defined as an unplanned procedure performed after the start of labour and labour as regular contractions at least every 10 min and cervical dilatation >3 cm; non reassuring fetal status (267), failure to progress in labour (189), malpresentation (34), prolonged second stage (15), other reasons (42)

^o^Indication: fetal breech presentation (14), cephalopelvic disproportion (10), nonprogress of labour (16), meconium stained liquor (25), fetal variable decelerations (20), scar tenderness (2), transverse lie (3), Type II decelerations (5), prolonged latent phase (2), previous lower segment cs with unfavorable cervix(3)

^p^Women with planned labour induction, were in early labour or were in active labour but had an epidural anaesthetic. Results were not reported separately for women with or without prepartal oxytocin administration

^q^In case of PPH, French national guidelines were applied

^r^Indications: previous CS (188), fetal distress (87), cephalopelvic disproportion (41), poor progress (30), failed induction of labour (14), breech (14), twins (11), other reasons (24)

CS: cesarean section; dl: decilitre; ED: effective dose; FHR: fetal heart rate; g: gram; GA: gestational age; h: hours; I1: intervention; I2: control intervention; im: intramuscular; imy: intramyometrial; intraop: intraoperatively; IUGR: intrauterine growth retardation; IU: international units; iv: intravenous; mg: milligram; min: minutes; ml: milliliter; mU: milliunit; n: number; NaCl: saline; non-RCT: non-randomized controlled trial; P: participants; postop: postoperatively; PPH: postpartum hemorrhage; prn: pro re nata; PROM: premature rupture of membranes; RCT: randomized controlled trial; s: seconds; SE: standard error; UK: United Kingdom; US: United States; μg: microgram; vs: versus

### Intrapartum cesarean delivery

From 36 studies identified, 8 studies (1705 patients) included solely women requiring intrapartum cesarean delivery, mainly because of labour arrest [26–33]. However, from these 8 studies, only 2 dose-response studies addressed the hypothesis whether parturients who receive intrapartum exogenous ocytocin are prone to a higher risk of PPH by evaluating uterine contraction after delivery [26, 27]: *(i)* Thereby, the dose-response study from Lavoie et al. investigated whether women who receive intrapartum exogenous oxytocin, and who subsequently undergo cesarean delivery for labour dystocia, need a higher estimated effective dose of oxytocin in 90% of women (ED90) compared with non-labouring parturients [27]. Thirty-two women participated in the labouring and 38 women in the non-labouring group. The reported oxytocin ED90 was significantly greater for the labouring group requiring cesarean delivery compared with that for the non-labouring group (mean difference: 28 Units/h [95% CI 26–29 Units/h, *P* < 0.001]). Furthermore, significantly more women in the labouring group compared to the non-labouring group required additional uterotonic agents (mean difference: 26% [95% CI 7–44%, *P* = 0.008]). *(ii)* The other dose-response study from Balki et al. estimated the minimum effective intravenous dose of oxytocin required for adequate uterine contraction after cesarean delivery for labour arrest, however, without using an active control group [26]. In this study, all 30 patients received oxytocin infusion at a mean of 9.8 ± 6.3 h before cesarean delivery. After delivery, oxytocin was administered as a slow intravenous bolus according to a coin up-down sequential allocation scheme. The minimum oxytocin ED90 required to produce adequate uterine response was estimated to be 3.0 U (95% CI 2.3–3.7 U). The authors concluded that women with intrapartum cesarean section for labour arrest require an oxytocin dose that is 9 times higher than previously reported after elective cesarean section in non-labouring women at term. However, the results of these dose-response studies are limited by comparing different patient populations without controlling for potential confounders such as women with increased risk of uterine atony, age, anticoagulant medication, general anaesthesia or multiple pregnancy. In addition, the authors used ‘satisfactory uterine contraction’ assessed by palpating the uterus as main outcome. Beside the fact that this outcome is subjective and not validated to estimate the effect of oxytocin on the uterus, it can not be excluded that due to a lack of blinding of the obstetricians, different thresholds for satisfactory uterine tone in women with prior labour compared with non-labouring women were used. Therefore, theses study results need to be interpreted with caution. The remaining 6 studies including women with intrapartum cesarean sections investigated different doses or application schedules of exogenous oxytocin or compared oxytocin to other uteronotics after cesarean delivery for PPH prophylaxis [28–32]. Whether the prepartal oxytocin status is a risk factor for atonic PPH could not be derived from these studies.

### Intrapartum and prelabour cesarean delivery

Our mapping also revealed that the majority of studies investigating PPH rates for different treatment groups are focused on prophylactic uterogenics and included women with different types of cesarean delivery (intrapartum and prelabour cesarean section). However, the PPH rates given in these studies were not stratified according to the indications for cesarean section. In addition, the prepartal oxytocin status was not considered as confounding factor by conducting subgroup analyses [34–63].

### Ongoing studies

The search for ongoing and unpublished studies identified 2 RCTs which may provide subgroup data estimating the blood loss and risk for PPH after cesarean section for labour arrest (ClinicalTrials.gov Identifier: NCT01869556 and NCT02794779). The estimated completion date for both studies is by the end of 2017. In addition, we identified HOLDS, a multicentre, double-blind randomised controlled trial to compare standard and high dose regimens of oxytocin for women with confirmed delay in the first stage of labour (http://www.isrctn.com/ISRCTN99841044). This trial randomises 1500 women and measure differences in rates and complications associated with cesarean section. If this study provides a subgroup analysis of PPH rates in those women requiring cesarean sections, important insights addressing the present research question will be given.

## Discussion

Current clinical practice guidelines for third stage oxytocin administration do not distinguish between women with prior oxytocin exposure and those without [64, 65]. This practice is also reflected by the current research in cesarean delivery, which showed that the majority of studies investigating oxytocin focus on prophylactic administration in the third stage of labour, without differing between the indications for cesarean section and, hence the prepartal oxytocin status (particularly when labour was induced or augmented). However, taking into account that labouring women requiring cesarean delivery constitute a relevant subset of patients - for whom originally spontaneous labour was anticipated - the lack of studies solely investigating this patient population is not surprising.

In total, our evidence map revealed that only 2 studies investigated the required oxytocin dose to prevent uterine atony after cesarean delivery for labour arrest. These dose-response studies support the hypothesis that labouring women exposed to exogenous oxytocin require a higher oxytocin dose after delivery than non-labouring women to prevent uterine atony after cesarean section. This finding is in accordance with the observations during spontaneous labour [24, 25]. However, the studies investigating this hypothesis in intrapartum cesarean delivery are all flawed by limitations of the study design as well as the outcome selection. Thus, the estimated oxytocin ED90 may be lower in labouring women than actually reported.

A limitation of our research work is that data could not be formally synthesized due to a lack of controlled studies evaluating the impact of ante partum oxytocin use on the risk of PPH in exogenous cesarean delivery. Nevertheless, the current scoping review describes key data of published studies investigating the routine use of oxytocin in caesarean delivery in their quantity, but also reveals major research gaps in this important but underrepresented obstetric area. It is obvious that research on this topic is mainly restricted to augmentation or induction of vaginal delivery [16, 66, 67] or the prophylactic use in the third stage of labour to prevent PPH [15, 68]. But such data would have been only relevant to address our research question, if they had allowed us to derive PPH rates for those women who required intrapartum cesarean delivery (e.g., data from subgroup analyses). A Cochrane Review from 2013 investigated oxytocin augmentation of labour in women with epidural analgesia for reducing operative deliveries [69]. This review provides rates for cesarean sections after oxytocin exposure in comparison to placebo, but whether there is a difference in PPH rates for women requiring cesarean section were also not provided. Taking into account that the current research does not provide us with rigorous controlled data to address our research question, access to individual and/or unpublished study data may be desirable [70]. Although overall progress has been made to obtain such study data, there are still major issues related to unrestricted data access. For example, released data may be incomplete, selective or not in compliance with the results reported in the corresponding publications. Facing these issues, we did not undertake efforts to include such data in our research work.

We believe that the current scoping review is important because it reveals that published research on oxytocin is restricted to the use for inducing or augmenting labour or for PPH prophylaxis, without considering women requiring intrapartum cesarean section – not even in subgroup analyses. Given the increasing rates in cesarean sections and labour inductions in many developed countries [71], well controlled studies are needed focusing on these parturient. Taking into account that different studies suggest that induction of labour increases the risk of postpartum hemorrhage and blood transfusion [24, 71], caution is needed when intrapartum cesarean section is performed, particularly when labour is induced or augmented, as it may result in increased maternal morbidity.

## Conclusions

Considering that research on the risk of PPH after oxytocin exposure is generally restricted to the use of PPH prophylaxis and vaginal births, future controlled studies should evaluate whether administration of oxytocin in cesarean delivery is associated with a higher risk of PPH taking into account that labouring and non-labouring women may show a difference response to exogenous oxytocin. In addition, these studies should control for known confounding factors associated with a higher risk of PPH such as multiple pregnancy, assisted reproductive technologies, general anaesthesia, type of uterine incision or advanced maternal age [6, 13, 14, 72]. As long as there is no evidence-based guideline available for labouring women requiring cesarean delivery, obstetricans and anaesthesiologists should always be aware of an increased risk of PPH due to prior oxytocin exposure in this patient population.

## Additional files

Additional file 1: PRISMA (2009) Checklist. (DOC 60 kb)

Additional file 2: Search Strategy in Medline (OvidSP). (DOCX 14 kb)

## Abbreviations

CI: Confidence Interval
ED: Effective Dose
IU: International Unit
OR: Odds Ratio
PPH: Postpartum Haemorrhage
PRISMA: Prefered Reporting Items for Systematic Reviews and Meta-Analyses
RCT: Randomized Controlled Trial
WHO: World Health Organization
