# Supplementary material for: Lack of controlled studies investigating the risk of postpartum haemorrhage in cesarean delivery after prior use of oxytocin: a scoping review
Source: BMC Pregnancy Childbirth. 2017 Nov 29;17:399. doi: 10.1186/s12884-017-1584-1 (PMC5708177; doi:10.1186/s12884-017-1584-1)
Supplement: Supplementary file 2 — Search Strategy in Medline (OvidSP). (DOCX 14 kb) [file 12884_2017_1584_MOESM2_ESM.docx]

Additional file 2. Search Strategy in Medline (OvidSP).

| # | Searches | Results |
| --- | --- | --- |
| 1 | exp Cesarean Section/ | 38130 |
| 2 | Vaginal Birth after Cesarean/ | 1313 |
| 3 | (caesar#an* or cesar#an*).ti,ab,kf. | 49945 |
| 4 | (deliver* adj3 abdom*).ti,ab,kf. | 856 |
| 5 | (c section* or csection*).ti,ab,kf. | 837 |
| 6 | vbac.ti,ab,kf. | 530 |
| 7 | sectio* caesar*.ti,ab,kf. | 71 |
| 8 | or/1-7 | 61420 |
| 9 | Oxytocin/ | 17664 |
| 10 | Receptors, Oxytocin/ | 1807 |
| 11 | (oxytocin* or ocytocin* or Syntocinon* or Pitocin*).ti,ab,kf,nm. | 24608 |
| 12 | or/9-11 | 24608 |
| 13 | 8 and 12 | 2284 |
| 14 | exp animals/ not humans/ | 4243660 |
| 15 | 13 not 14 | 2249 |
| 16 | remove duplicates from 15 | 2221 |
| 17 | (retracted publication or "retraction of publication").pt. | 8888 |
| 18 | 16 not 17 | 2221 |
